# Traitement chirurgical d'une luxation palmaire carpo-métacarpienne à propos d'un cas

**DOI:** 10.11604/pamj.2015.22.160.8009

**Published:** 2015-10-20

**Authors:** Adil El Alaoui, Mouhcine Sbiyaa, Badr Alami, Amine Mezzani, Amine Marzouki, Fawzi Boutayeb

**Affiliations:** 1 Service de Chirurgie Orthopédique et Traumatologique (A) du Centre Hospitalier Universitaire Hassan II de Fès, Fès, Maroc

**Keywords:** luxation, carpo-métacarpienne, embrochage

## Abstract

Les luxations carpo-métacarpiennes sont des lésions rares, les auteurs rapportent un cas de luxation carpo-métacarpienne palmaire du cinquième doigt, traité en urgence par réduction et stabilisation par embrochage à foyer fermé. Une immobilisation postopératoire par une attelle intrinsèque plus a été réalisée pendant six semaines, avec une rééducation à partir de la quatrième semaine. Le résultat fonctionnel était satisfaisant.

## Introduction

Les luxations carpo-métacarpiennes des doigts sont des lésions rares. Le premier cas a été décrit par Rivington en 1873 [1]. Le diagnostic est suspecté cliniquement et confirmé par la radiologie. Le traitement consiste à faire une réduction en urgence, une stabilisation par des broches en cas de lésion instable et une immobilisation plâtrée complémentaire.

## Patient et observation

**Figure 1:**
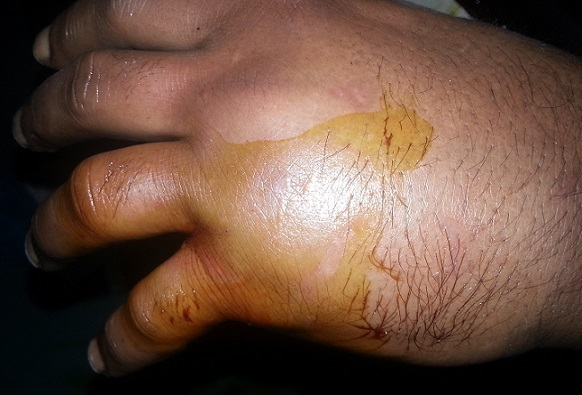
Aspect de la main montrant un important oedème et deformation du cinquième doigt

**Figure 2:**
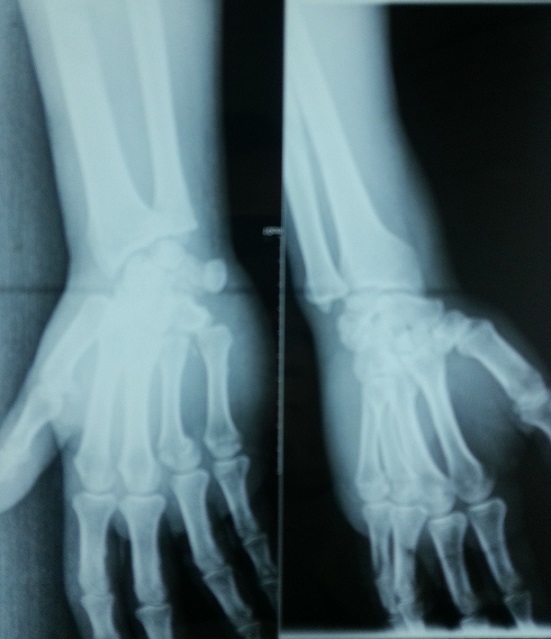
Les radiographies de face et de profil de la main droite objectivaient une luxation carpo-métacarpienne palmaire, associée à une fracture du col du quatrième métacarpe et une fracture de la base du premier phalange du cinquième doigt

**Figure 3:**
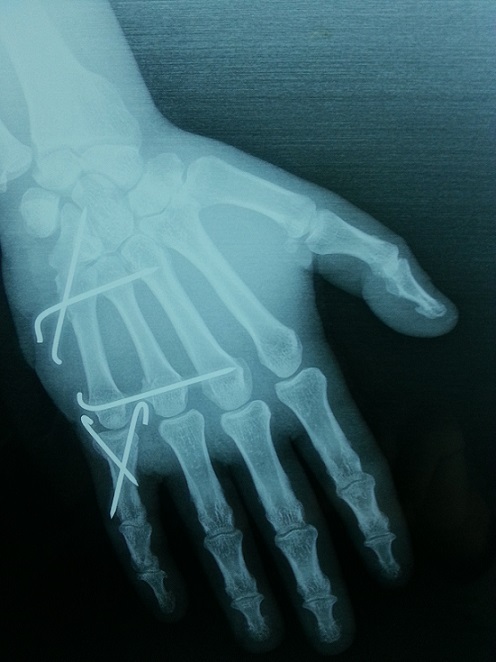
La radiographie de la main droite en post-opératoire après ostéosynthèse de la luxation et les fractures associées par embrochage

Un patient âgé de 38 ans maçon de profession, droitier, sans antécédents pathologiques particuliers est admis aux urgences à la suite d'une chute d'une échelle d’ une hauteur estimée à 3 mètres avec réception sur le bord cubital de la main droite. Il s'agissait d'un traumatisme fermé de la main droite occasionnant chez lui douleur et impotence fonctionnelle. L'examen clinique a objectivé un œdème et déformation de la face palmaire de la main droite (Figure 1). Les radiographies de face et de profil (Figure 2) de la main droite objectivaient une luxation carpo-métacarpienne palmaire, pure, complète, associée à une fracture du col du quatrième métacarpe et une fracture de la base de la première phalange du cinquième doigt. Notre patient a été opéré en urgence, sous anesthésie locorégionale, garrot à la racine du membre. La réduction carpo-métacarpienne était facile à foyer fermé suivi d'une synthèse par embrochage (Figure 3). Le poignet a été immobilisé par une attelle en position intrinsèque plus pendant une durée de 6 semaines, dés lors on a réalisé l'ablation des broches. La rééducation active des doigts a été entreprise dès la quatrième semaine postopératoire en gardant l'attelle entre les séances de rééducation. À 10 mois de recul, le résultat fonctionnel était satisfaisant avec une bonne force musculaire et une récupération complète de la mobilité du poignet en flexion-extension et des doigts. Le travail a été repris trois mois plus tard.

## Discussion

L'articulation carpo-métacarpienne est une articulation très stable [2, 3], tous les auteurs s'accordent pour souligner l'extrême violence nécessaire pour désorganiser l'emboîtement articulaire [4]. Ceci rend la luxation carpo-métacarpienne des doigts une lésion rare. Elle intéresse l'adulte jeune. Les traumatismes très violents comme les accidents de la circulation sont les principaux responsables. Cependant, les traumatismes de plus faible intensité comme les coups de poing, évoqués lors d'une luxation des métacarpiens mobiles [5]. Si le diagnostic de ce type de lésion est fait en urgence sur un cliché radiographique de la main et du poignet de profil strict le pronostic est meilleur bien que l'interprétation des clichés radiographiques soit parfois difficile. Il est primordial de réaliser une incidence de profil strict montrant le sens du déplacement des bases métacarpiennes, une incidence oblique dégageant les métacarpiens mobiles ou fixes et une incidence de face. Par ailleurs, certains auteurs recommandent une étude tomodensitométrique complémentaire [2]. La réduction par manœuvres externes avec brochage percutané est un bon traitement en l'absence de compression vasculo-nerveuse associée La stabilisation par broches des interlignes carpo-métacarpiens peut être oblique, intra médullaire ou en croix [5]. Le résultat de ces luxations carpo-métacarpiennes traitées en urgence est bon et laisse peu de séquelles [6–8]. Plusieurs complications ont été rapportées dans la littérature, comme la persistance de douleurs résiduelles de la main, la diminution de la force de préhension, les subluxations et les déplacements secondaires [9]. Par ailleurs, Lawlis et Gunther [5] ont signalé que les patients qui ont une luxation des quatre articulations carpo-métacarpiennes ont de meilleurs résultats que ceux qui présentent une luxation des deuxième et troisième rayons [5, 10]. Les 4^e^ et 5^e^ articulations carpo-métacarpiennes doivent être embrochées en légère flexion pour conserver l'incurvation de l'arche métacarpienne [11, 12].

## Conclusion

Les luxations carpo-métacarpiennes des doigts longs sont des lésions rares, souvent associées à des fractures du carpe ou des os de la main. Un grand nombre passe inaperçues soit à cause d'un examen sommaire mal fait ou rentrant dans le cadre d'un polytraumatisme. Sous réserve d'un traitement urgent et correct, elles sont de bon pronostic.
